# Bacterial Inactivation of Wound Infection in a Human Skin Model by Liquid-Phase Discharge Plasma

**DOI:** 10.1371/journal.pone.0024104

**Published:** 2011-08-29

**Authors:** Paul Y. Kim, Yoon-Sun Kim, Il Gyo Koo, Jae Chul Jung, Gon Jun Kim, Myeong Yeol Choi, Zengqi Yu, George J. Collins

**Affiliations:** Department of Electrical and Computer Engineering, Colorado State University, Fort Collins, Colorado, United States of America; Institut de Pharmacologie et de Biologie Structurale, France

## Abstract

**Background:**

We investigate disinfection of a reconstructed human skin model contaminated with biofilm-formative *Staphylococcus aureus* employing plasma discharge in liquid.

**Principal Findings:**

We observed statistically significant 3.83-log10 (p<0.001) and 1.59-log10 (p<0.05) decreases in colony forming units of adherent *S. aureus* bacteria and 24 h *S. aureus* biofilm culture with plasma treatment. Plasma treatment was associated with minimal changes in histological morphology and tissue viability determined by means of MTT assay. Spectral analysis of the plasma discharge indicated the presence of highly reactive atomic oxygen radicals (777 nm and 844 nm) and OH bands in the UV region. The contribution of these and other plasma-generated agents and physical conditions to the reduction in bacterial load are discussed.

**Conclusions:**

These findings demonstrate the potential of liquid plasma treatment as a potential adjunct therapy for chronic wounds.

## Introduction

A biofilm is an aggregate of microorganisms embedded in a self-produced matrix of extracellular polymeric substances. According to the National Institutes of Health, biofilms account for over 80% of microbial infections in the body. Common examples include infections of the urinary tract, middle ear, and paranasal sinuses. Biofilms are responsible for life-threatening diseases such as pneumonia in cystic fibrosis patients and endocarditis and have also been implicated in the prevention of wound healing in chronic wounds. Bacteria within biofilms are notoriously resistant to antimicrobial agents due to restricted antibiotic diffusion through the matrix, slow growth rates, and induction of a resistant phenotype [1]. Because biofilms can persist in 20 to 1000 times the concentrations of drugs that inhibit planktonic bacteria [2]–[4], the optimum treatment against biofilms usually involves additional physical disruption to achieve removal [5].

Plasma, often referred to as the fourth state of matter, has emerged as an attractive germicidal tool capable of physically destroying microorganisms with minimal heat damage to the substrate. Plasma generates a rich mixture of reactive oxygen species, ozone, and ultraviolet radiation (UV), each of which are known to inactivate bacteria [6]. Various groups including our own have studied bacterial inactivation and biofilm removal by atmospheric pressure non-thermal plasma on surfaces such as agar or surgical implants, yet in-situ studies on living tissues remain limited. Since in-vitro tests on inanimate objects tend to overestimate antimicrobial activity [7], [8] and the same properties that kill microorganisms may be harmful to human tissue, we studied plasma sterilization on EpiDerm engineered human skin constructs as a first step.

Our preliminary observations showed that argon gas plasma could sterilize a lawn of *Staphylococcus aureus* on agar within 30 s, but was significantly less effective against a much lighter inoculum on EpiDerm. In view of these conflicting results using plasma discharge in gas, we explored bacterial inactivation using plasma discharge in liquid. Histological analysis suggested that the irregular surface of the dermal compartment might be shielding some of the bacteria from the gas plasma. We hypothesized that liquid plasma may have greater accessibility to these crevices and provide greater concentration of bactericidal species due to higher radical densities. Below we report for the first time, bacterial inactivation employing plasma discharge in liquid in a reconstructed human skin model.

Plasma discharges in liquid (hereafter called simply ‘liquid plasma’) have been intensely investigated during the last two decades due to their potential in diverse applications such as selective polymer surface modification [9], synthesis of nano-materials [10], water purification [11], and orthopedic surgery [12]. The numerous liquid plasma configurations and the complex physics of discharges lie beyond the scope of this paper; the interested reader is referred to a review by Bruggeman and Leys [13].

## Materials and Methods

### Liquid Plasma Device and Setup

**Figure 1:**
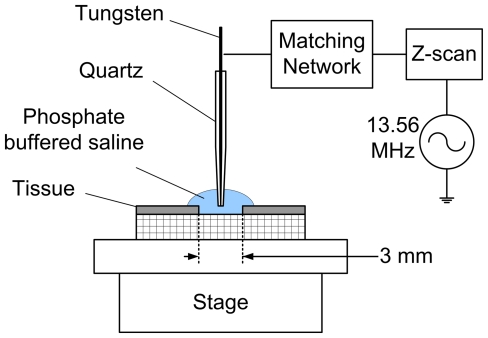
Schematic cross-sectional illustration of the liquid plasma device and experimental setup. The tissue is an EpiDermFT full-thickness human skin model consisting of an epidermis (gray) and dermis (grid pattern) with a central 3 mm diameter wound. Tissue culture insert is not depicted for clarity.

The liquid plasma electrode consists of a tungsten needle sheathed in quartz with the needle tip exposed. This electrode is connected to a 13.56 MHz radio frequency (RF) power supply (Cesar, Advanced Energy, Fort Collins, CO) through an impedance matching network and Z-Scan RF probe (Advanced Energy) for real-time measurement of RF power and impedance as illustrated in Figure 1.

### Full Thickness Reconstructed Human Skin Model

EpiDermFT tissues (EFT-400-WH) with a 3 mm diameter wound induced by a biopsy punch were purchased from MatTek Corporation (Ashland, MA), along with antibiotic-free maintenance medium and MTT tissue viability kits. The EpiDermFT construct is a full-thickness (700–900 µm), differentiated model of the human dermis and epidermis consisting of human epidermal keratinocytes and human dermal fibroblasts. The construct closely parallels human tissue and is useful for assessing dermal toxicity. Tissue samples were maintained at 37°C in a humidified atmosphere of 5% CO_2_ throughout the study.

### Bacterial Culture and Inactivation

The wound was contaminated with a 20 µl inoculum (7.4×10^8^ CFU/ml) of biofilm-formative *Staphylococcus aureus* (subsp. *aureus* Rosenbach, ATCC 12600) grown overnight in thioglycollate medium enriched with vitamin K_1_ and hemin. Bacteria were allowed to grow in the wound for 3 h for attachment and adhesion or 24 h for biofilm development prior to treatment. Following these predetermined incubation periods, all tissue samples were rinsed three times with phosphate buffered saline (PBS) to remove nonadherent bacteria. Tissue samples were then covered with 300 µl of PBS and placed on an XYZ stage to align the center of the wound with the liquid plasma electrode at a gap distance of 2 mm. Pulsed RF output power was applied at 10 W, 10 Hz, 10% duty cycle for 1 min, 2 min, 3 min, or 5 min in the case of 3 h grown bacteria samples and for 3 min in the case of 24 hr grown biofilm samples. Control tissue samples (0 min) were kept in PBS for 5 min with no RF power applied. After treatment, the PBS was removed and the center of each wound was collected by a 2 mm diameter biopsy punch and vortexed in 7 ml of 0.85% saline. Serial dilutions were spread on blood agar plates (trypticase soy agar with 5% sheep blood). The plates were incubated at 37°C for 24 h to determine bacterial colony counts.

Although we do not present herein evidence of biofilm formation, the strain of *S. aureus* (ATCC 12600) used in this study has previously been found to be a strong biofilm former [14]. *S. aureus* biofilms are routinely formed by culturing for ∼24 h under static conditions described by Christensen et al. [15], as in this study, and have also been studied as early as 6 h to 8 h after inoculation [16]–[19].

### Histology

Tissue samples were removed from the tissue culture inserts, fixed in 10% formalin, dehydrated through a series of graded alcohols, cleared in xylene, and embedded in paraffin. Next, the embedded tissues were cut into 5 µm sections and stained using hematoxylin and eosin (H&E). Finally, a pathologist who remained blinded to the treatments assessed the specimens under light microscopy for abnormalities.

### Tissue Viability

The tissue samples were rinsed with PBS and treated with liquid plasma as described above but without any bacteria. Tissue samples were then incubated with the MTT reagent (2 mg/ml) for 3 h. Formazan crystals were subsequently extracted overnight for determination of optical density at 570 nm. As a positive control, 200 µl of 1% Triton X-100 was applied to the apical tissue surface for 4 h prior to the MTT assay.

### Nitric Oxide

Nitric oxide has a very short half-life in water. The stable end products of nitric oxide oxidation were measured using a nitrate/nitrite colorimetric assay kit based on the Griess reaction, purchased from Cayman Chemical (Ann Arbor, Michigan).

### Optical Emission Spectroscopy

The light emitted by the plasma discharge was collected with a fiber optic cable and recorded in the 200 nm to 1000 nm range using an Ocean Optics HR4000CG spectrometer (Dunedin, FL) to identify active species.

### Plasma and Liquid Temperature

Additional emission spectra collected using a 307.1 nm optical filter with a bandwidth of 10 nm were compared with theoretical spectra generated by LIFBASE software to calculate plasma temperature. Liquid (PBS) temperature was measured by a Neoptix fiber optic temperature sensor (Québec, Canada). The optic fiber tip was located 2 mm below the plasma electrode.

### Statistical Analysis

Data were analyzed by Student's t-test or by one-way ANOVA with Dunnett's post test as appropriate using GraphPad Prism Version 5.0c (San Diego, CA). A value of p<0.05 was considered to indicate statistical significance. All data presented are mean ± SEM of at least three independent experiments.

## Results

**Figure 2:**
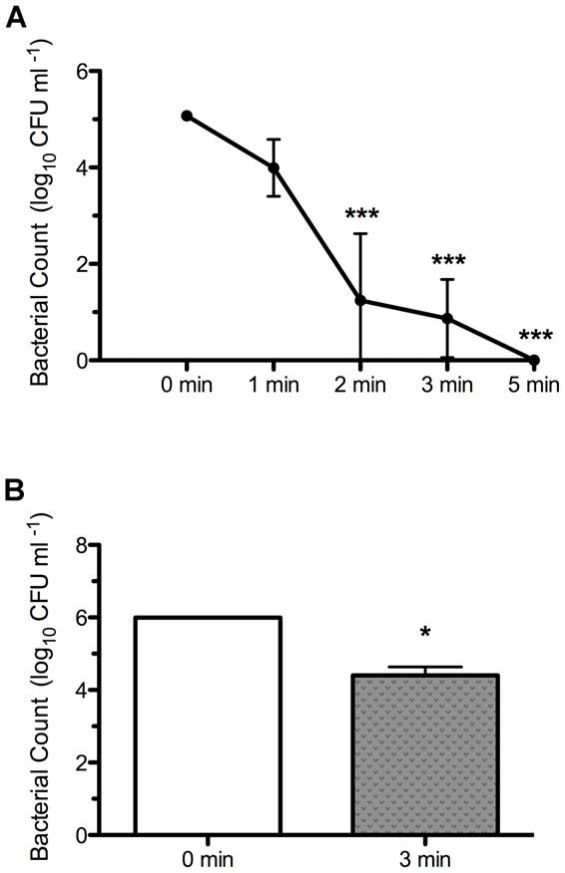
Bacterial inactivation. Bacterial counts of EpiDermFT human skin model incubated for (A) 3 h or (B) 24 h with biofilm-formative *Staphylococcus aureus* and treated with liquid plasma for indicated durations. (A) After 3 h bacteria incubation, statistically significant (p<0.001) reduction in colony forming units was observed with 2 min treatment compared with the control (0 min). (B) After 24 h bacteria incubation, statistically significant (p<0.05) reduction in colony forming units was observed with 3 min treatment compared with the control (0 min).

We observed a statistically significant (p<0.001) 3.83-log_10_ decrease in colony forming units (CFUs) with 2 min liquid plasma treatment compared with the control (Figure 2). Although 5 min treatment resulted in zero detectable CFUs, there were no statistically significant differences in inactivation rates for treatment durations longer than 2 min. When bacteria were allowed to grow in the wound for 24 h, the inactivation rate diminished to 1.59-log_10_ with 3 min treatment, but this was nonetheless a statistically significant (p<0.05) reduction compared with the control.

**Figure 3:**
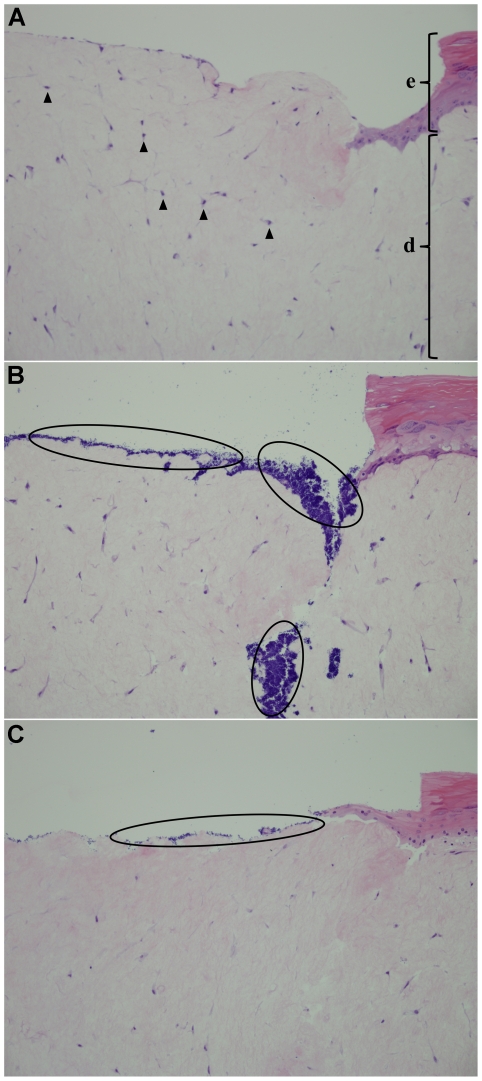
Histological analysis. Hematoxylin and eosin-stained histological sections of EpiDermFT human skin model (A) without bacteria, (B) incubated with bacteria for 3 h, and (C) incubated with bacteria for 3 h and treated with liquid plasma for 2 min. A focal ulceration where the epithelium (e) is lacking can be observed. The dermis (d) is populated by fibroblasts (arrowheads) and purple-stained *S. aureus* (circles) on the surface and within the tissue. 200X magnification.

The histological sections consisted of keratinized, stratified squamous epithelium and underlying dermis as shown in Figure 3. The wound from the biopsy punch appeared as a focal ulceration completely lacking the epithelium. Examination of tissue samples incubated with *S. aureus* revealed abundant cocci, adherent to the surface and forming colonies deep within the tissue (Figure 3b). Liquid plasma-treated samples showed decreased number of bacteria both at the surface and within the tissue. No morphological changes to the tissue were noted with 1 min and 2 min treatments (Figure 3c). Decreased cellularity of the dermal stroma was evident and the tissues were slightly edematous with 3 min and 5 min treatments.

**Figure 4:**
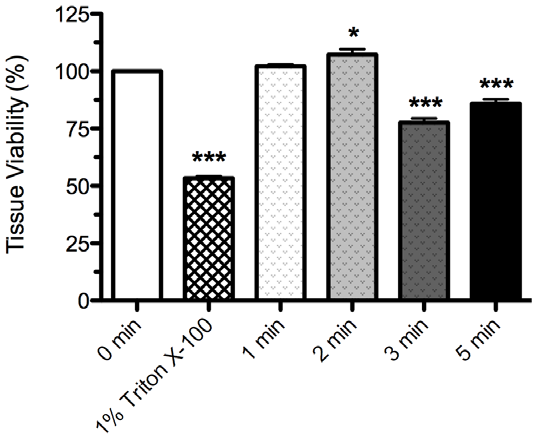
Tissue viability. MTT assay of EpiDermFT human skin model treated with liquid plasma for indicated durations and with 1% Triton X-100 for 4 h as a positive control. Data are expressed as percent of absorbance measured in control tissues (0 min). Statistically significant (p<0.001) reduction in cell viability was observed with Triton X-100 exposure and 3 min and 5 min liquid plasma treatments compared with untreated control (0 min).

Liquid plasma treatment for 1 min duration did not affect tissue viability as determined by MTT assay as illustrated in Figure 4. Increasing the treatment duration beyond 2 min decreased tissue viability compared with the control, by 22% at 3 min and by 14% at 5 min durations. These results are in agreement with the histological analysis. Our tissue viability data carry the caveat that plasma species are unlikely to penetrate the full thickness of the tissue sample and measuring the viability of the entire tissue is likely to underestimate tissue damage. Application of 1% Triton X-100 as positive control resulted in an ET_50_ (exposure time required to decrease viability by 50%) of ∼4 h. The discrepancy between the observed ET_50_ value and the 8.6±1.2 h previously reported by MatTek [20] is likely due to the compromised barrier function of the wound model used in the present study.

**Figure 5:**
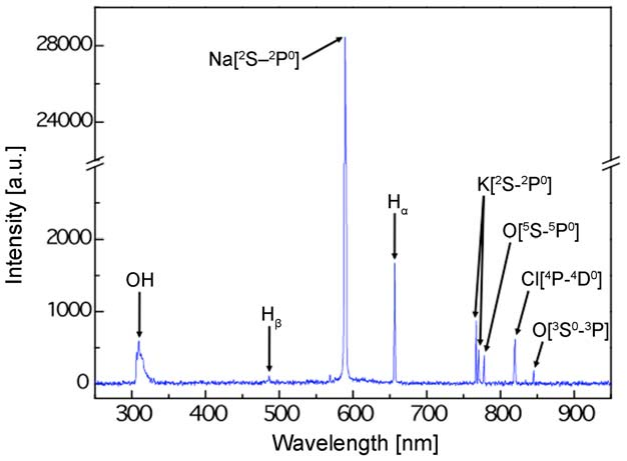
Optical emission spectrum. A typical spectrum collected from the liquid plasma discharge in phosphate buffered saline at 10 W RF power with spectral lines labeled with their corresponding species.

The plasma was characterized by means of optical emission spectroscopy (OES) to identify possible excited chemical species that may influence the tissue and bacteria. OES relies on the principle that an excited species emits photons at a characteristic wavelength as the electrons return to the ground state. Spectral analysis of these emissions from plasma discharge in PBS revealed that atomic Na, K, and Cl lines from the buffer salts dominate the excited species as shown in Figure 5. Emissions from atomic oxygen radicals were detected at 777 nm and 844 nm. UV radiation (between 40 nm and 400 nm) emitted by the plasma was also observed. The contribution of these and other plasma-generated agents to the bactericidal effect is discussed below.

**Figure 6:**
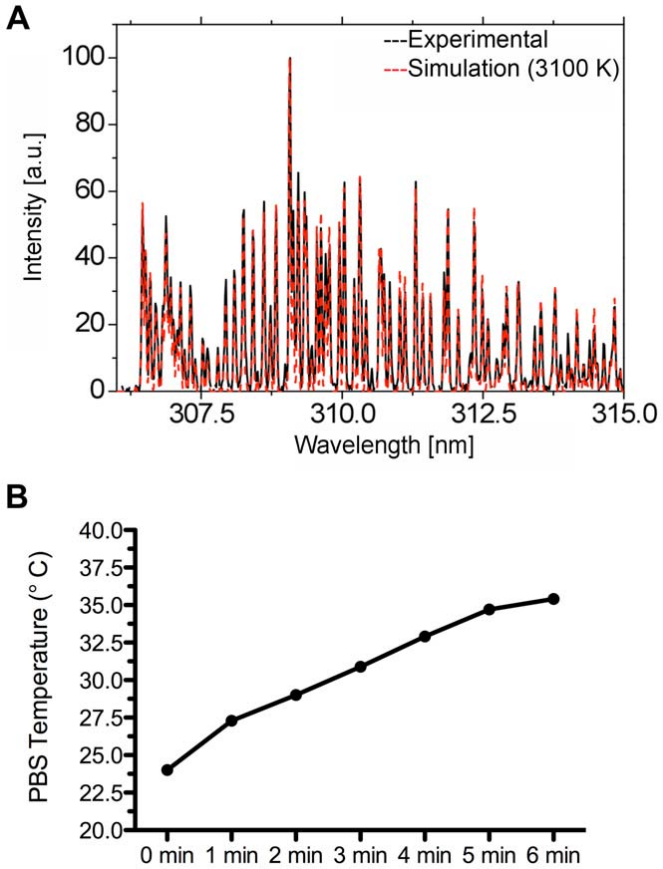
Thermal characterization of plasma. Spectra in the 306 nm to 315 nm region containing the OH (A–X) band (A). Data obtained by LIFBASE simulation (in red) matches the experimentally observed data (in black) and corresponds to a calculated rotational temperature of 3100 K. The temperature of the PBS (B) obtained by fiber optic thermometer 2 mm below the electrode as a function of time.

The temperature of the plasma calculated by LIFBASE simulation was 3100 K. The simulated spectra (in red) perfectly matched the actual observed spectra (in black). The temperature of the PBS increased from 24°C to 35.4°C after 6 min of plasma operation (Figure 6).

## Discussion

In the present study, a reconstructed human skin model of a wound contaminated with biofilm-formative *S. aureus* was disinfected using a liquid-phase discharge plasma driven by 10 W RF power. When tissue samples were incubated with bacteria for 3 h, the bacterial inactivation rate reached a maximum statistically significant value of 99.985% with a 2 min treatment. In the 24 h biofilm culture, 97.43% reduction in bacterial load was observed with a 3 min treatment. Plasma treatment for 3 min or longer appeared to injure the tissue, as tissue viability was reduced with these longer treatment durations. In contrast to the minor impact on tissue viability of 2 min treatment with liquid plasma driven by 10 W RF power, 2 min treatment at 20 W dramatically decreased tissue viability by 84% relative to control (data not shown). These preliminary data underline the importance of balancing plasma parameters in order to inactivate bacteria without damaging the tissue and the associated need for well-controlled drive electronics and accurate monitoring electronics in the feedback loops.

We observed a statistically significant 7% increase in tissue viability with 2 min liquid plasma treatment (at 10 W). The biological relevance of this effect is not clear. Some studies have reported NO-induced cell proliferation following exposure to air plasma discharge, which can produce high concentrations of NO from atmospheric nitrogen [21], [22]. In our liquid plasma discharge, however, the concentration of NO was below the limit of detection (less than 1 µM). The effect may be mediated by other external factors influencing cell growth or reflect an increase in enzymatic activity of the mitochondria. Further studies to explore liquid plasma-assisted wound healing might be merited.

Consistent with the results from the tissue viability assay, histological examination revealed no microscopic abnormalities with 1 min and 2 min liquid plasma treatment, but decreased cellularity was apparent with longer treatment durations. Although the histology does not indicate whether the bacteria are alive or dead, purple-stained cocci were abundant in tissue samples incubated with *S. aureus*. Consistent with the bacterial colony counts, far fewer bacteria were apparent in tissue samples treated with liquid plasma. Washing away of loosely adherent bacteria by the liquid cannot account for the relative reduction in bacterial load since all tissue samples, including control, were thoroughly rinsed with PBS just prior to treatment. Rough, porous surfaces (such as those found in the dermal compartment) have been shown to favor bacterial adhesion [23.24]. Extracellular matrix proteins produced by dermal fibroblasts such as collagen [25], elastin [26], and fibronectin [27], [28] are also known to promote *S. aureus* adhesion via a family of protein adhesins. Through these nonspecific and specific interactions, bacteria can adhere strongly to the tissue surface. This would be true in particular for the tissue samples incubated with bacteria for 24 h since bacterial adhesion becomes more irreversible as the adhering organisms excrete extracellular polymeric substances during biofilm development.

In plasma discharges in water, intense shockwaves emanating from the expanding plasma channel have long been studied [29.30] and exploited for drinking water and wastewater treatment [31]. We cannot state with certainty that shockwaves do not play some role here in the physical removal or killing of adherent bacteria, but it is an unlikely scenario due to the relatively low energy (<100 mJ) of our plasma pulse. Shockwaves form from high-energy (≥1 kJ) pulse discharges [32]. Our own high-speed camera studies of gas bubble dynamics at the electrode tip confirm slow expansion of sub-mm-sized bubbles in the order of 10^2^ µs. We hypothesize that the gas bubbles carry bactericidal agents produced at the electrode to the tissue surface. The modest pressures generated may act to drive these agents into the dermal recesses where the bacteria are observed to colonize (Figure 3b). This could explain in part the greater bacterial inactivation obtained using liquid plasma as opposed to argon gas plasma (10 W 13.56 MHz RF power), with which a 5 min treatment resulted in a 2.1-log_10_ reduction (data not shown). Capillary action may also play a role in delivering liquid plasma agents to otherwise inaccessible bacteria.

Plasma sterilization studies typically invoke reactive chemical species and UV radiation as the two major plasma-generated agents responsible for bacterial inactivation. A common theme in both gas and liquid plasma is the generation of reactive chemical species and UV radiation. One advantage of liquid versus gas plasma is that the greater density of liquid provides orders of magnitude higher concentration of reactants for the formation of reactive species. Spectral analysis of the liquid plasma discharge indicates the presence of highly reactive atomic oxygen radicals (777 nm and 844 nm) as well as OH bands in the UV region (Figure 5). Reactive oxidant species are well known to damage and kill bacteria [33], [34] and have been postulated to play a major role in bacterial inactivation by gas plasma [35]–[37]. The OH is presumably produced from the plasma-driven dissociation of H_2_O [38]. While recombination of OH is known to generate H_2_O_2_, the H_2_O_2_ concentration (in the order of ppm) would be negligible from a disinfection standpoint in the present plasma discharge.

Its short penetration depth limits UV radiation to inactivating surface-borne bacteria. Thus, UV is likely to play a minor role when bacteria lie within the irregular surface of the dermis, shielded from the radiation. The calculated rotational temperature of the plasma at the electrode is 3100 K (Figure 6a). This heat energy may drive chemical reactions in the PBS at the electrode tip but given the small volume of plasma in an open system, the temperature of the PBS where the sample was situated remained below 37°C throughout the study (Figure 6b). Therefore, thermal effects are not likely to impact the bacteria or tissue. The role of sodium excited states through sensitized reactions and radiation trapping we judge is also significant but is the subject of future studies.

These results represent a single treatment with liquid plasma. A multidose treatment regimen may be useful in managing biofilm-related wounds given the remarkable resistance of biofilm infections to a standard course of antibiotic treatment. Further characterization of the inactivation mechanisms and studies of more mature biofilms will help increase our understanding of liquid plasma as a possible treatment modality for chronic wounds.

## References

[1] Mah TFC, O′Toole GA (2001). Mechanisms of biofilm resistance to antimicrobial agents.. Trends Microbiol.

[2] Nickel JC, Russia I, Wright JB, Costerton JW (1985). Tobramycin resistance of *Pseudomonas aeruginosa* cells growing as a biofilm on urinary catheter material.. Antimicrob Agents Chemother.

[3] Evans RC, Holmes CJ (1987). Effect of vancomycin hydrochloride on Staphylococcus epidermidis biofilm associated with silicone elastomer.. Antimicrob Agents Chemother.

[4] El-Azizi M, Rao S, Kanchanapoom T, Khardori N (2005). In vitro activity of vancomycin, quinupristin/dalfopristin, and linezoid against intact and disrupted biofilms of staphylococci.. Ann Clin Microbio Antimicrob.

[5] McDonnell GE (2007). Antisepsis, disinfection, and sterlization: types, action, and resistance..

[6] Kong MG, Kroesen G, Morfill G, Nosenko T, Shimizu T et al (2009). Plasma medicine: an introductory review.. New J Phys.

[7] Maillard JY, Messager S, Veillon R (1998). Antimicrobial efficacy of biocides tested on skin using an ex-vivo test.. J Hosp Infect.

[8] Messager S, Goddard PA, Dettmar PW, Maillard JY (2001). Determination of the antibacterial efficacy of several antiseptics tested on skin by an ‘ex-vivo’ test.. J Med Microbiol.

[9] Joshi R, Schulze R, Meyer-Plath A, Wagner MH, Friedrich JF (2009). Selective surface modification of polypropylene using underwater plasma technique or underwater capillary discharge.. Plasma Process Polym.

[10] Sano N, Wang H, Alexandrou I, Amaratunga GAJ (2001). Synthesis of carbon ‘onions’ in water.. Nature.

[11] Chang JS, Dickson S, Guo Y, Urashima K, Emelko MB (2008). Electrohydraulic discharge direct plasma water treatment processes, in Advanced Plasma Technology..

[12] Vankov A, Palanker D (2007). Nanosecond plasma-mediated electrosurgery with elongated electrodes.. J Appl Phys.

[13] Bruggeman P, Leys CJ (2009). Non-thermal plasmas in and in contact with liquids.. Phys D: Appl Phys.

[14] Møretrø T, Hermansen L, Holck AL, Sidhu MS, Rudi K (2003). Biofilm formation and the presence of the intercellular adhesion locus *ica* among staphylococci from food and food processing environments.. Appl Environ Microbiol.

[15] Christensen GD, Simpson WA, Younger JJ, Baddour LM, Barrett FF (1985). Adherence of coagulase-negative staphylococci to plastic tissue culture plates: a quantitative model for the adherence of staphylococci to medical devices.. J Clin Microbiol.

[16] Amorena B, Gracia E, Monzón MM, Leiva J, Oteizab C (1999). Antibiotic susceptibility assay for *Staphylococcus aureus* in biofilms developed *in vitro*.. J Antimicrob Chemother.

[17] Johnson M, Cockayne A, Williams PH, Morrissey JA (2005). Iron-responsive regulation of biofilm formation in Staphylococcus aureus invovles Fur-dependent and Fur-independent mechanisms.. J Bacteriol.

[18] Sambanthamoorthy K, Schwartz A, Nagarajan V, Elasri MO (2008). The role of *msa* in *Staphylococcus aureus* biofilm formation.. BMC Microbiol.

[19] Resch A, Leicht S, Saric M, Pásztor L, Jakob A (2006). Comparative proteome analysis of *Staphylococcus aureus* biofilm and planktonic cells and correlation with transcriptome profiling.. Proteomics.

[20] Cannon CL, Neal PJ, Kubilus J, Klausner M, Harbell JW (1994). Presented at symposium, “Alternatives in the Assessment of Toxicity; Theory & Practice”..

[21] Shekhter AB, Kabisov RK, Pekshev AV, Kozlov NP, Perov YL (1998). Experimental and clinical validation of plasmadynamic therapy of wounds with nitric oxide.. Bull Exper Biol Med.

[22] Kalghatgi SU, Fridman A, Friedman G, Morss-Clyne A (2009). Cell proliferation following non-thermal plasma is related to reactive oxygen species induced fibroblast growth factor-2 release..

[23] Taylor RL, Verran J, Lees GC, Ward AJP (1998). The influence of substratum topography on bacterial adhesion to polymethyl methyacrylate.. J Mater Sci: Mater Med.

[24] Scheuerman TR, Camper AK, Hamilton MA (1998). Effects of substratum topography on baterial adhesion.. J Col Interf Sci.

[25] Speziale P, Raucci G, Visai L, Switalksi LM, Timpl R (1986). Binding of collagen to *Staphylococcus aureus* Cowan 1.. J Bacteriol.

[26] Park PW, Roberts DD, Grosso LE, Parks WC, Rosenbloom J (1991). Binding of elastin to *Staphylococcus aureus*.. J Biol Chem.

[27] Vaudax PE, Waldvogel FA, Morgenthaler JJ, Nydegger UE (1984). Adsorption of fibronectin onto polymethylacylate and promotion of *Staphylococcus aureus* adherence.. Infect Immunol.

[28] Herrmann M, Vaudaux PE, Pittit D (1988). Fibronectin, fibrinogen, and laminin act as mediators of adherence of clinical staphylococci isolates to foreign material.. J Infect Dis.

[29] Brandt B, Edebo L, Hedén CG, Hjortzberg-Nordlund B, Selin I (1962). The effect of submerged electrical discharges on bacteria.. Teknisk Vetenskaplig Forskining.

[30] Edebo L, Selin I (1968). The effect of the pressure shockwave and some electrical quantities in the microbicidal effect of transient arcs in aqueous systems.. J Gen Microbiol.

[31] Locke BR, Sato M, Sunka P, Hoffman MR, Chang JS (2006). Hydraulic discharge and nonthermal plasma for water treatment.. Ind Eng Chem Res.

[32] Bogomaz AA, Goryachev VL, Remennyi AS, Rutberg FG (1991). The effectiveness of a pulsed electrical discharge in decontaminating water.. Solv Technol Phys Lett.

[33] Miller RA, Britigan BE (1999). Role of oxidants in microbial pathophysiology.. Clin Microbiol Rev.

[34] Storz G, Imlay JA (1999). Oxidative Stress.. Curr Opin Microbiol.

[35] Laroussi M, Leipold F (2004). Evaluation of the roles of reactive species, heat, and UV radiation in the inactivation of bacterial cells by air plasmas at atmospheric pressure.. Int J Mass Spectrom.

[36] Deng X, Shi J, Kong MG (2006). Physical mechanisms of inactivation of *Bacillus subtilis* spores using cold atmospheric plasmas.. IEEE Trans Plasma Sci.

[37] Perni S, Shama G, Hobman JL, Lund PA, Kershaw CJ (2007). Probing bactericidal mechanisms induced by cold atmospheric plasmas with *Escherichia coli* mutants.. Appl Phys Lett.

[38] Koo IG, Lee WM (2006). Hollow-cathode based electrical discharge in atmospheric pressure water vapor at wide range of temperature.. Jpn J Appl Phys.

